# Epidemiological analysis of pesticide poisoning cases in Zibo City, Shandong Province, China: a decade-long study (2014–2023)

**DOI:** 10.1186/s12889-026-28329-6

**Published:** 2026-07-16

**Authors:** Ning Li, Guangyong Liu, Huali Duan, Fengjiang Song, Yitang Liu

**Affiliations:** 1 Zibo Center for Disease Control and Prevention, Zibo, Shandong 255026 China; 2 Zibo First Hospital, Zibo, Shandong 255000 China

**Keywords:** Pesticide poisoning, Epidemiological characteristics, Mortality risk factors, Case fatality rate

## Abstract

**Background:**

Pesticides are extensively employed across agricultural, public health, and industrial sectors, yielding significant benefits in crop protection and food security. However, their overuse and misuse have escalated pesticide poisoning into a pressing global public health issue—particularly in developing countries such as China. As the world’s largest pesticide consumer, China continues to experience a disproportionately high burden of pesticide-related morbidity and mortality, even following the nationwide phase-out of highly hazardous compounds. Alarmingly, intentional ingestion of pesticides remains the most common method of suicide in the country. Shandong Province—the leading agricultural producer in China—offers a strategically relevant setting for epidemiological investigation. Within it, Zibo City stands out as a representative case: it integrates both traditional and modern farming systems and operates a well-established, comprehensive surveillance system for pesticide poisoning, thereby providing robust empirical grounding for analyzing the distribution, determinants, and trends of pesticide poisoning.

**Methods:**

A retrospective epidemiological study was conducted on pesticide poisoning cases reported in Zibo City between 2014 and 2023. Case data were extracted from the Occupational Disease and Health Hazard Factors Monitoring Information System—a nationally standardized subsystem of China’s Integrated Disease Prevention and Control Information System—while population denominators were obtained from the official Zibo Statistical Yearbooks (2014–2023 editions). A structured case database was developed in Microsoft Excel, and all statistical analyses were performed using R 4.5.2 Categorical variables were compared using chi-square tests (or Fisher’s exact test where expected cell counts were < 5), and binary logistic regression was employed to identify independent predictors of in-hospital mortality; results are presented as adjusted odds ratios (aOR) with 95% confidence intervals (CI), with statistical significance set at *P* < 0.05. Case definitions encompassed demographic characteristics, exposure circumstances, pesticide classification (e.g., organophosphates, carbamates), clinical severity, treatment outcomes, and occupational attribution—specifically distinguishing between occupational and non-occupational poisoning.

**Results:**

A total of 762 pesticide poisoning cases were identified between 2014 and 2023 in Zibo City, including 400 males (52.5%) and 133 fatalities, yielding an overall case fatality rate (CFR) of 17.6%. The mean age of cases was 55.10 ± 20.04 years, with an average annual incidence of 1.63 per 100,000 population. The 65-75-year age group accounted for the largest proportion of cases (20.5%), and CFR increased monotonically with advancing age. Females exhibited a significantly higher CFR (18.8%) than males (16.5%). Non-occupational intentional ingestion—predominantly self-poisoning—constituted the leading exposure mechanism (68.9% of all cases) and carried the highest CFR (23.6%), whereas no fatalities were observed among occupationally exposed individuals (CFR = 0%). Among pesticide classes, herbicides demonstrated the highest CFR (48.3%), followed by insecticides (14.6%); organophosphorus compounds represented 84.3% of all insecticide-related poisonings, with dichlorvos being the most frequently implicated active ingredient. Temporally, the annual incidence of pesticide poisoning rose steadily over the study decade, peaking in 2023 (*n* = 147) and reaching its nadir in 2015 (*n* = 30), while the CFR declined progressively. Seasonally, cases were concentrated between March and October: occupational and intentional poisonings peaked during June–August, whereas accidental poisonings clustered in April–September. Geographically, Linzi, Zichuan, and Gaoqing Districts reported the highest case counts; conversely, Huantai, Yiyuan, and Boshan Districts recorded elevated CFRs. Multivariable binary logistic regression identified four independent predictors of in-hospital mortality: age ≥ 45 years (with CFR risk increasing incrementally with age), female sex (aOR = 1.69, 95% CI: 1.08–2.72), herbicide exposure (aOR = 1.79, 95% CI: 1.16–2.80), and non-occupational intentional ingestion (aOR = 2.71, 95% CI: 1.31–6.37). Comparative analysis with the preceding 2006–2013 period revealed a declining trend in the proportion of organophosphorus pesticide poisonings and a concurrent rise in herbicide-related incidents.

**Conclusions:**

Zibo’s pesticide poisoning features a notably higher CFR than other Chinese regions, with rising incidence yet falling CFR reflecting partial treatment success and unoptimized prevention, and non-occupational intentional ingestion stands as the core issue for local control. Age, gender, pesticide type and poisoning cause are key mortality factors, and the poisoning presents distinct demographic and geographical distribution characteristics driven by rural aging, social pressure and regional medical-economic gaps, for which targeted prevention and control strategies are proposed accordingly. This study has certain limitations in data sources and research scope, so future research needs to improve data collection and expand research coverage to offer more scientific support for relevant policies.

**Trial registration:**

Clinical trial number: Not applicable. This is a retrospective observational epidemiological study without any health-related interventions assigned to participants, which does not meet the WHO/ICMJE definition of a clinical trial, so trial registration is not required.

**Supplementary Information:**

The online version contains supplementary material available at https://doi.org/10.1186/s12889-026-28329-6.

## Introduction

In a broad context, pesticides encompass chemically synthesized compounds, natural products, biotechnological agents, and their formulations, which are employed to prevent, eradicate, or manage harmful organisms in agricultural and forestry settings, as well as to modulate the metabolic, developmental, and reproductive processes of both plants and pests [1]. These substances are extensively utilized across diverse domains—including agriculture, forestry, animal husbandry, domestic hygiene, pest management, and disease control—and have yielded substantial benefits. Nevertheless, the excessive and inappropriate application of pesticides has progressively elevated pesticide poisoning to a matter of global concern, particularly in developing nations such as China and India [2].

The heterogeneity of pesticide classes—ranging from organophosphates and carbamates to neonicotinoids and biopesticides—gives rise to clinically diverse poisoning presentations, including acute cholinergic toxicity [3], delayed neuropathy [4], chronic neurocognitive deficits [5], endocrine disruption [6], and carcinogenic potential [7]. As a major agricultural economy, China accounts for approximately 1.4 million metric tons of annual pesticide use—the highest globally [8]. Although national regulatory actions—including the phase-out of 46 highly hazardous pesticides since 2007—have reduced availability of the most toxic agents, pesticide poisoning incidents remain persistently high, particularly in rural areas. Critically, intentional ingestion of pesticides constitutes the leading method of suicide in China, responsible for an estimated 50% of all suicide deaths nationwide [9].

The Chinese government has prioritized the monitoring of pesticide poisoning incidents, establishing a national surveillance system for occupational disease hazards with mandatory online reporting via the China Disease Prevention and Control Information System in July 2006 [10]. As a leading agricultural province, Shandong consistently ranks first in China by the total output value of its agriculture, forestry, animal husbandry, and fishery sectors. Zibo, representing a model for agricultural modernization in Shandong, exhibits distinct patterns in pesticide production and application. To elucidate the epidemiological characteristics of pesticide poisoning in this region, the present study provides a comprehensive analysis of reported cases in Zibo City spanning a ten-year period from 2014 to 2023.

## Methods

### Study area

Zibo is located in the central part of Shandong Province, within the transitional zone between the mountainous terrain of central Shandong and the North China Plain. This unique geographical context fosters a notable diversity of agricultural output. The region’s agricultural profile is marked by the significant cultivation of wheat, millet, vegetables, and fruit crops, all of which require consistent pesticide use. In parallel, Zibo has achieved substantial progress in digital agriculture, having established six digital agricultural industrial chains and completed 115 major projects in digital agriculture and rural development. The co-occurrence of traditional and modern agricultural practices, along with a comprehensive pesticide poisoning surveillance system, makes Zibo a particularly representative region for this study.

### Data sources

The data were obtained from individual case reports of pesticide poisoning incidents recorded in Zibo City. These reports were submitted between 2014 and 2023 by healthcare institutions at various administrative levels through the “Occupational Disease and Health Hazard Factors Monitoring Information System,” a subsystem of the China Information System for Disease Control and Prevention. This system serves as China’s national-level core information platform for the surveillance, reporting, and management of occupational diseases and workplace health hazards. It enables comprehensive case reporting and data acquisition for pesticide poisoning incidents in Zibo City. Clinical details of pesticide poisoning cases were documented by healthcare professionals during medical consultations, with each report undergoing a multi-level review process to ensure data reliability. The final dataset was exported from the aforementioned system, maintained by the Chinese Center for Disease Control and Prevention. Corresponding population statistics were sourced from the Zibo City Statistical Yearbooks(This yearbook provides annual statistical data on Zibo City’s national economy and social development, serving as our primary source for demographic information pertaining to the city.) spanning the years 2014 to 2023.

This study extracted and analyzed all requisite variables from the reported data, including demographic characteristics, pesticide poisoning types, and pesticide classifications. A standardized set of criteria and classification rules was consistently applied across all variables.

### Case definition

Case records encompass the patient’s date of birth, sex, geographic location of poisoning incident, etiology of poisoning, time of diagnosis, pesticide classification, clinical outcome, and the primary healthcare facility responsible for treatment. The pesticide poisoning addressed in this article refers to acute intoxication resulting from dermal, inhalational, or oral exposure to pesticides during production, storage, application, and related occupational activities. Poisoning causes are classified as either occupational or non-occupational exposures.

### Data analysis

This study established a case database in Microsoft Excel and performed all statistical analyses using R version 4.5.2. Categorical variables were compared between groups using the chi-square test. A binary logistic regression model was fitted with in-hospital mortality due to pesticide poisoning as the outcome variable (death = 1; survival = 0), with the logit link function applied for multivariate modeling. Covariates—including age, sex, cause of poisoning, and types of pesticides—were selected based on clinical relevance and univariate screening (*p* < 0.10). Results are reported as aORs with 95% confidence intervals. All statistical tests were two-sided, and a p-value < 0.05 was considered statistically significant.

## Results

### Essential information on pesticide poisoning

Between 2014 and 2023, Zibo City reported a cumulative total of 762 cases of pesticide poisoning, including 400 male cases, accounting for 52.5% of the total. The average age of the affected population was 55.10 ± 20.04 years. During the same period, there were 134 fatal cases. The annual incidence rate of pesticide poisoning was 1.63 per 100,000 people, and the overall CFR reached 17.6% (Table 1).

**Table 1:** Distribution of Pesticide Poisoning Cases in Zibo City, 2014–2023

Variable	Incident cases		Death cases	Case fatality rate (%)	*P* value
	*N*	%			
Total	762	100	134	17.6	
Age groups					0.0246
< 15	31	4.1	0	0.0	
15 ~ < 25	40	5.3	6	15.0	
25-~<35	64	8.4	12	18.8	
35-<45	74	9.7	8	10.8	
45 ~ < 55	155	20.3	21	13.6	
55 ~ < 65	124	16.3	28	22.6	
65 ~ < 75	156	20.5	33	21.2	
≥ 75	118	15.5	26	22.0	
Gender 0.464					
Male	400	52.5	66	16.5	
Female	362	47.5	68	18.8	
Cause of poisoning <0.001					
Occupational poisoning	39	5.1	0	0	
Non-Occupational(accident)	198	26.0	10	5.1	
Non-Occupational(suicide)	525	68.9	124	23.6	
Types of pesticides					<0.001
Insecticide	596	78.2	87	14.6	
Bactericide	0	0.0	0	0	
Rodenticide	44	5.8	1	2.3	
Herbicide	87	11.4	42	48.3	
Biochemical Pesticides	16	2.1	2	12.5	
Others	19	2.5	2	10.5	

Pesticide poisoning incidents affect people of all age groups. Among them, the age group of 65–75 years has the highest number of cases, accounting for 20.5% (*n* = 156), followed by the age group of 45–55 years, accounting for 20.3% (*n* = 155). As shown in Fig. 1, the incidence rate of pesticide poisoning shows an upward trend with age. Further analysis by poisoning type reveals that cases of occupational pesticide poisoning are mainly concentrated in the age range of 35–75 years; cases of accidental ingestion in daily life are mainly distributed in the age group of ≥ 45 years and the younger age group of < 15 years; the age - distribution characteristics of cases of self - ingestion (suicide) in daily life are consistent with those of overall poisoning cases. Among the various categories of poisoning cases, intentional self-ingestion accounted for the largest proportion (68.9%) and was associated with the highest mortality rate (23.6%), exceeding those of occupational pesticide exposure and unintentional ingestion in daily life.

**Fig. 1:**
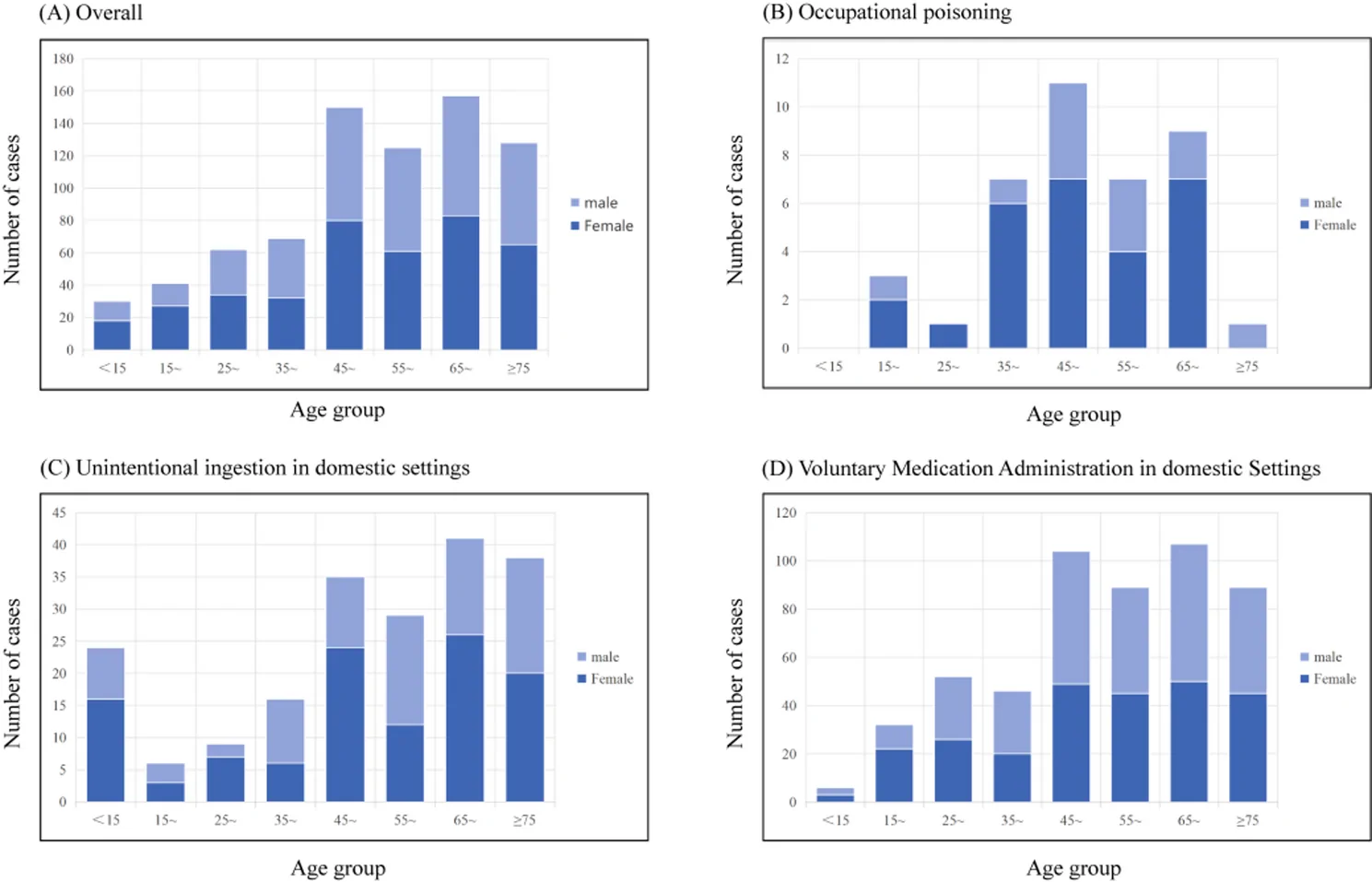
Demographic distribution of pesticide poisoning cases by age and sex in Zibo City, 2014–2023. (**A**) Overall（**B**）Occupational poisoning（**C**）Unintentional ingestion in domestic settings（**D**）Voluntary Medication Administration in domestic Settings

Fatalities were distributed across all age groups aged 15 years and above, with a notable upward trend in CFR as age increased. Regarding gender disparities, the CFR among females (18.8%) was higher than that among males (16.5%). By pesticide category, herbicide poisoning exhibited the highest CFR at 48.3%, followed by insecticide poisoning with a CFR of 14.6%. (**A**) Overall（B）Occupational poisoning（C）Unintentional ingestion in domestic settings（D）Voluntary Medication Administration in domestic Settings

## Annual distribution of pesticide poisoning cases

As illustrated in Fig. 2, the incidence rate of pesticide poisoning in Zibo City exhibited an overall upward trend from 2014 to 2023. The year with the highest number of cases was 2023 (*n* = 147), while the lowest was recorded in 2015 (*n* = 30). Despite the increasing trend in case numbers over the study period, the CFR demonstrated a general decline.

**Fig. 2:**
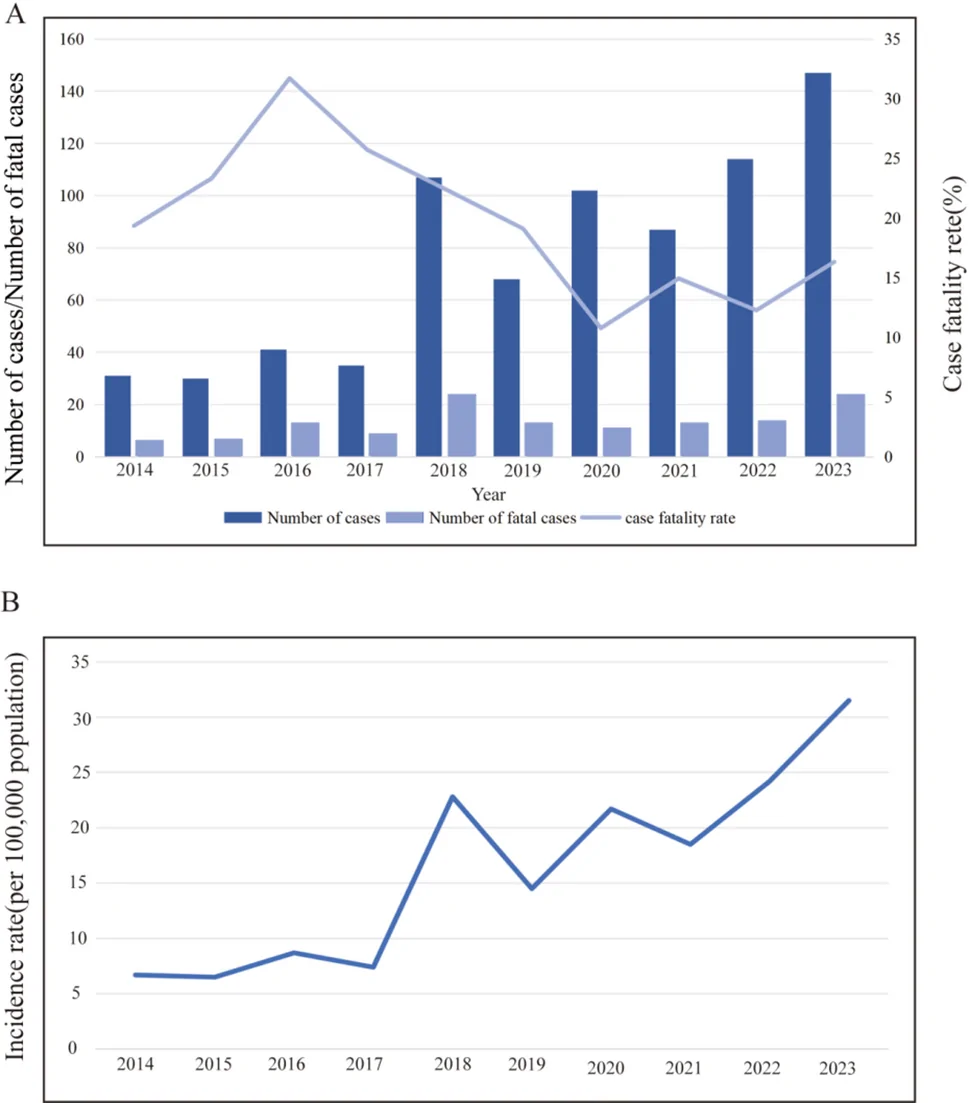
Temporal Trends in Pesticide Poisoning Cases, Incidence Rates, Mortality, and Case Fatality Rates in Zibo City, 2014–2023

As illustrated in Fig. 3a, the annual number of occupational poisoning cases remained relatively stable from 2014 to 2023. In contrast, both accidental and suicidal poisoning cases exhibited a sustained upward trend over the same period. While the incidence rate of occupational poisoning fluctuated year-to-year, it consistently remained low; conversely, the incidence rates of accidental and suicidal poisoning increased markedly. Notably, the CFR for occupational poisoning was zero across all ten years. For suicidal poisoning, the CFR remained comparatively high but demonstrated a clear downward trajectory throughout the study period.

**Fig. 3:**
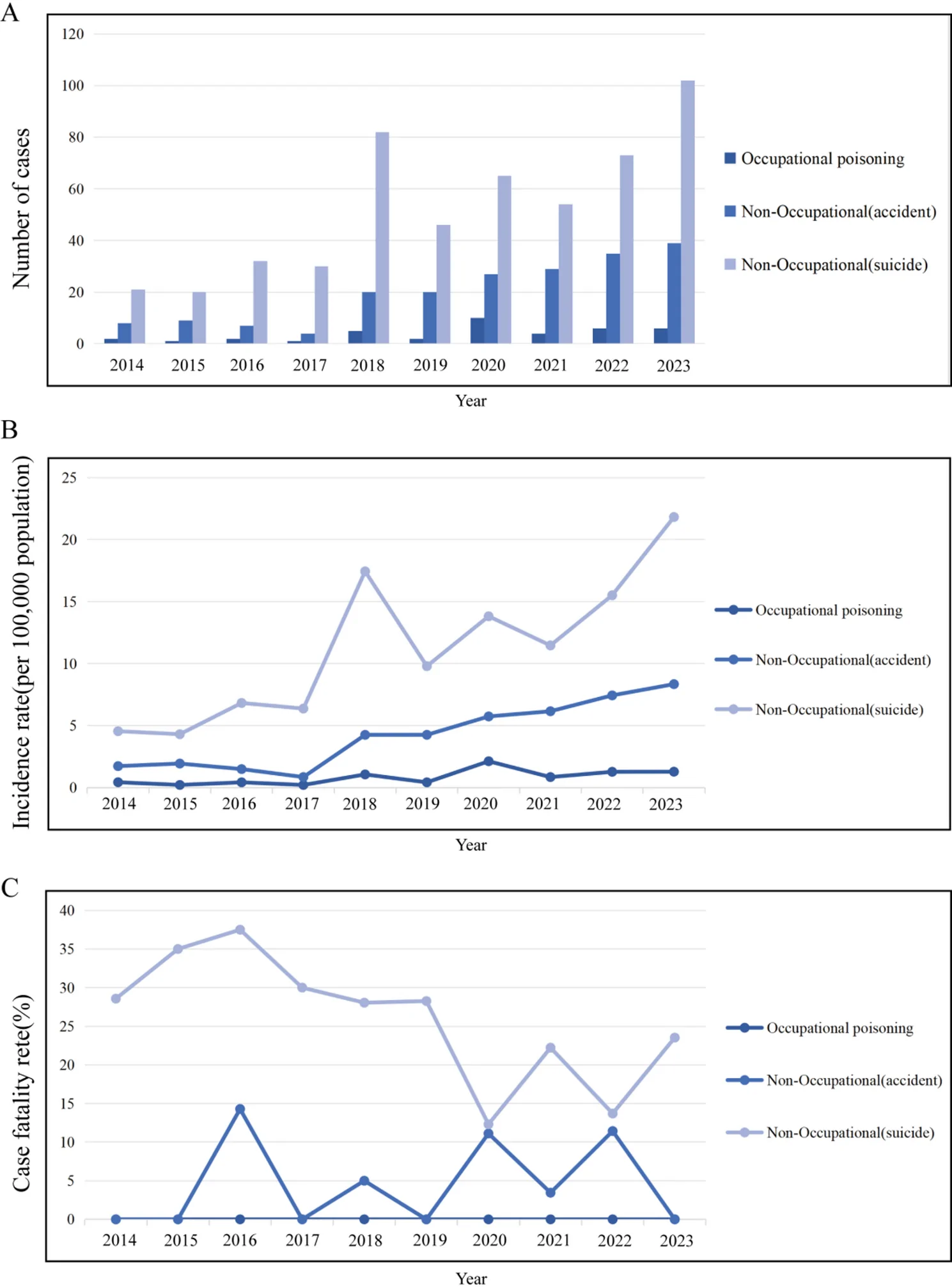
Annual Distribution of Pesticide Poisoning Cases, Incidence Rates, and Case-Fatality Rates by Etiologic Agent: (**a**) Case Count; (**b**) Incidence Rate per 100,000 Population; (**c**) Case-Fatality Rate (%)

## Monthly distribution of pesticide poisoning cases

As illustrated in Fig. 4, pesticide poisoning incidents exhibit a distinct temporal pattern, with the majority occurring between March and October. Among these, occupational poisoning cases demonstrate strong seasonality, with the highest proportion of annual cases observed in July and August. The temporal distribution of intentional (suicidal) poisoning closely resembles that of occupational poisoning, also peaking between June and August. In contrast, accidental poisoning cases are predominantly concentrated from April to September, displaying a fluctuating trend overall (Fig. 4a).

**Fig. 4:**
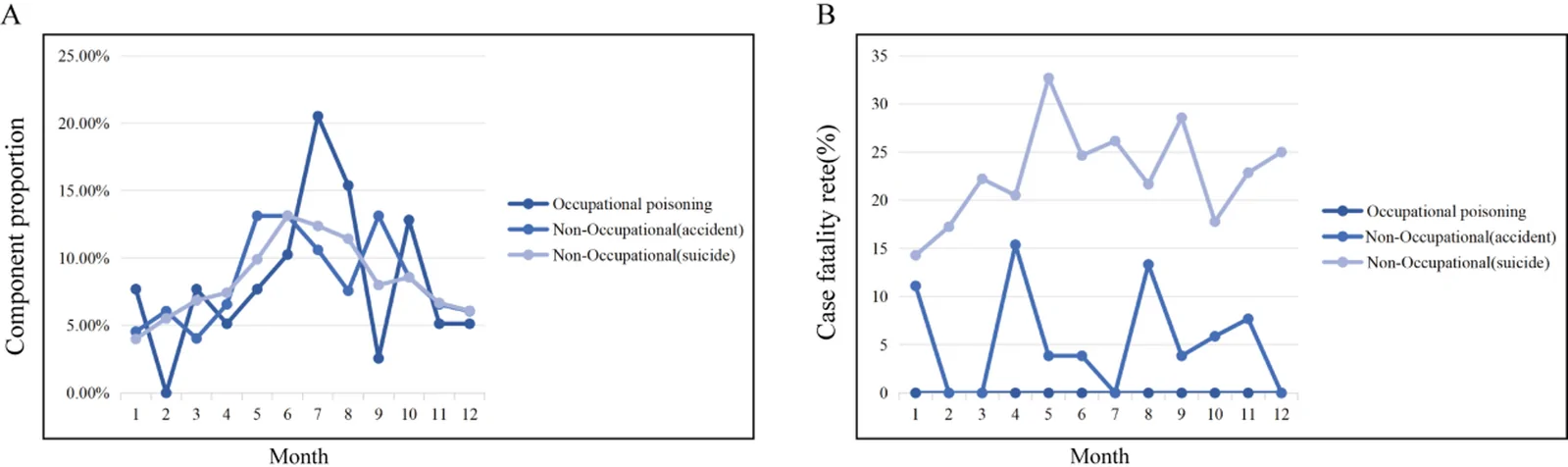
Monthly Distribution of Proportional Morbidity and Case-Fatality Rate by Etiologic Agent of Pesticide Poisoning: (**a**) Proportional Morbidity (%); (**b**) Case-Fatality Rate (%)

Meanwhile, as shown in Fig. 4b, the case-fatality rate of intentional (suicidal) poisoning remains consistently high, primarily concentrated between May and September. The case-fatality rate for accidental poisoning exhibits fluctuations and occurs mostly from April to November. In comparison, the case-fatality rate for occupational poisoning remains at a low level throughout the year without significant seasonal variation.

### Regional distribution of pesticide poisoning cases

A marked disparity exists in the reported incidence of pesticide poisoning across various districts and counties of Zibo City. The cases are predominantly concentrated in Linzi District, followed by Zichuan District and Gaoqing District. In terms of fatality rates, Huantai District, Yiyuan County, and Boshan District exhibit relatively higher proportions, which does not entirely align with the geographic distribution of case numbers (Fig. 5).

**Fig. 5:**
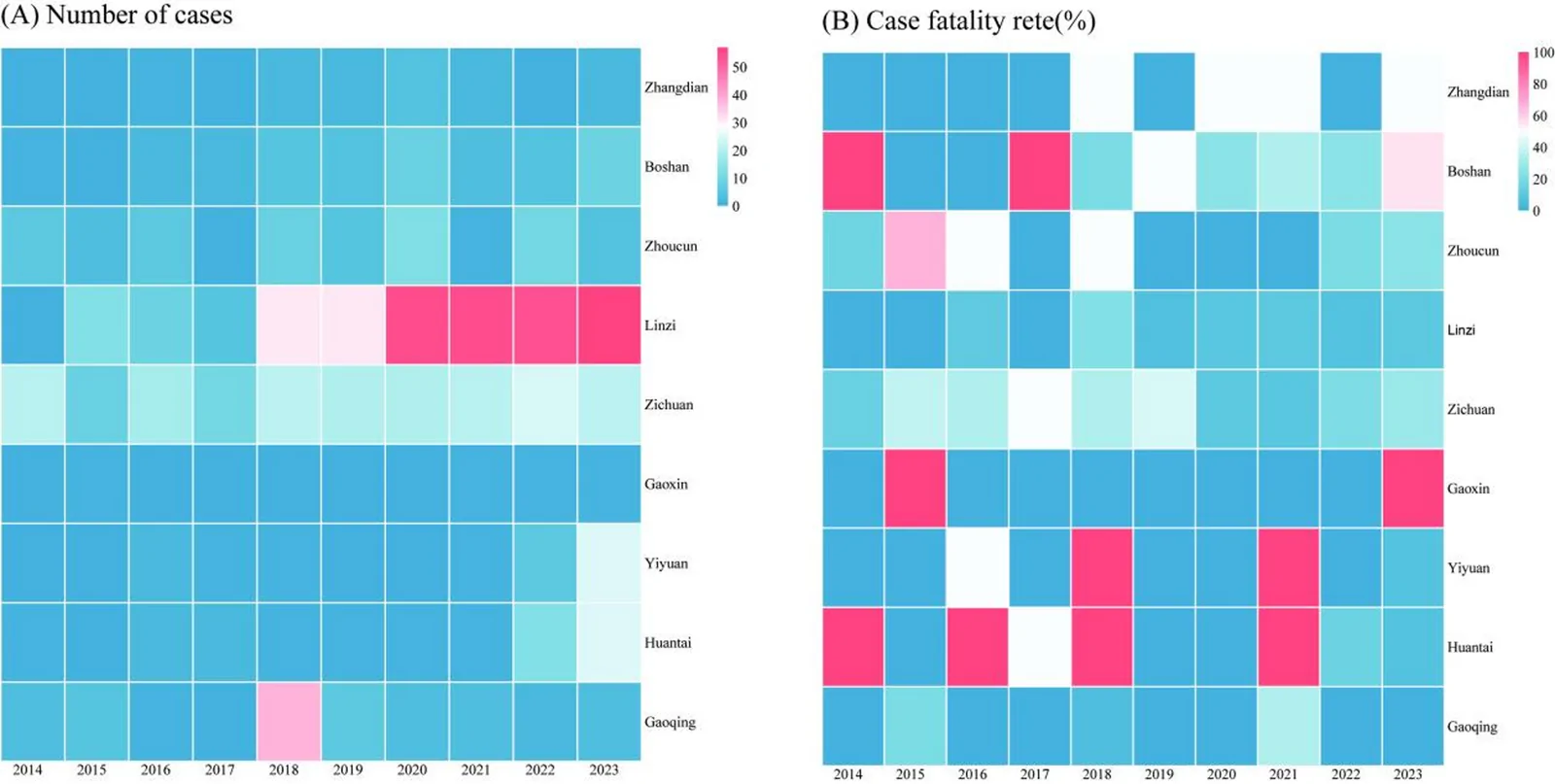
Regional Distribution of Pesticide Poisoning Cases and Case-Fatality Rates in Zibo City: (**a**) Case Count; (**b**) Case-Fatality Rate (%)

To assess regional disparities in pesticide poisoning mortality, we aggregated case and death counts from 2014 to 2023 by administrative region, constructed a region–outcome contingency table, and applied the Pearson chi-square test. The analysis revealed statistically significant heterogeneity in mortality rates across regions (χ² = 67.09, *P* < 0.001). However, interpretation of these differences requires caution: in several regions, cumulative case counts were extremely low—rendering mortality estimates highly susceptible to stochastic variation and thereby limiting their epidemiological reliability.

### Distribution of pesticide poisoning types

Among 762 cases of pesticide poisoning, insecticides constituted the predominant category, with 596 cases accounting for 78.2%. This was followed by herbicides and rodenticides, which represented 11.4% and 5.8% of cases, respectively. Within the insecticide group, organophosphorus pesticides were the leading cause of poisoning, comprising 84.3% of cases. Among these, dichlorvos intoxication was the most frequent, representing 75.0% of organophosphorus poisoning cases.

Regarding CFR, herbicide poisoning exhibited the highest mortality at 48.3%, followed by insecticide poisoning with a fatality rate of 15.0%. Among individual pesticides, paraquat, other herbicides, and dichlorvos demonstrated elevated fatality rates of 71.7%, 22.0%, and 17.9%, respectively.

A comparative analysis of pesticide poisoning patterns between the periods 2014–2023 and 2006–2013 revealed a statistically significant difference in the distribution of poisoning types (χ² = 29.590, *P* < 0.05). Notably, the proportion of organophosphorus pesticide poisoning showed a declining trend, whereas the incidence of herbicide and other pesticide poisonings exhibited an upward trend.

### Influencing factors of pesticide-related mortality

Logistic regression analysis revealed that all age groups aged 45 and above exhibited a significantly higher risk of pesticide-related mortality compared to the under-25 group (*P* < 0.05), with the risk progressively increasing with age (Table 2). Females demonstrated a markedly elevated mortality risk relative to males, with an aOR (95% CI) of 1.69(1.08 ~ 2.72). Pesticide type significantly influenced mortality risk: compared to insecticides, exposure to herbicides (aOR = 1.79, 95% CI: 1.16–2.80) and other pesticide types (aOR = 1.82, 95% CI: 1.04–3.14) was associated with a statistically significant increase in mortality risk. No significant differences were observed for other pesticide categories. Regarding exposure scenarios, intentional ingestion in non-occupational settings posed a substantially higher mortality risk compared to occupational pesticide poisoning, with an aOR (95% CI) of 2.71 (1.31–6.37).

**Table 2:** Determinants of Pesticide-Related Mortality in Zibo City, 2014–2023

Variable	aORs (95%CIs)	*P* value
Age groups		
<25	Ref.	
25 ~ < 35	2.5(0.91 ~ 7.6)	0.086
35 ~ < 45	1.31(0.43 ~ 4.19)	0.631
45 ~ < 55	1.7(0.69 ~ 4.81)	0.277
55 ~ < 65	3.16(1.32 ~ 8.83)	0.016
65 ~ < 75	2.91(1.23 ~ 8.02)	0.023
≥ 75	2.86(1.19 ~ 7.98)	0.027
Gender		
Male	Ref.	
Female	1.69(1.08 ~ 2.72)	0.026
Cause of poisoning		
Occupational poisoning	Ref.	
Non-Occupational(accident)	1.96(0.96 ~ 4.56)	0.088
Non-Occupational(suicide)	2.71(1.31 ~ 6.37)	0.012
Types of pesticides		
Insecticide	Ref.	
Rodenticide	0.78(0.33 ~ 1.67)	0.547
Herbicide	1.79(1.16 ~ 2.8)	0.009
Biochemical Pesticides	0.92(0.14 ~ 3.46)	0.914
Others	1.82(1.04 ~ 3.14)	0.033

## Discussion

This population-based retrospective study comprehensively characterized the epidemiological patterns and determinants of pesticide poisoning in Zibo City, Shandong Province, from 2014 to 2023, with the objective of informing evidence-based prevention and intervention strategies. A total of 762 confirmed cases were identified during the study period, yielding an CFR of 17.6%. This CFR substantially exceeds those reported in contemporaneous regional studies: 5.3% in Zhejiang Province [10], 5.5% in Jiangsu Province [11], and 11.8% in Quzhou City [12]. Temporal analysis revealed a statistically significant upward trend in annual incidence, whereas the CFR declined progressively, suggesting improvements in acute clinical management. However, the persistent rise in incidence underscores critical gaps in primary prevention—particularly in pesticide access regulation, safe storage practices, and community-level health education. Notably, the 2020 surveillance data exhibited an anomalous pattern: a 28.7% increase in reported cases coincided with stable fatal outcomes, resulting in a transient CFR reduction to 13.9%. This deviation is attributable to pandemic-related disruptions—including heightened public health surveillance intensity, altered healthcare-seeking behavior, and temporary shifts in occupational exposure patterns—and must therefore be explicitly accounted for when interpreting secular trends.

By poisoning etiology, non-occupational cases constituted the predominant exposure category (82.4%), underscoring their primacy as a public health priority for targeted prevention and clinical intervention. This pattern aligns with findings from contemporaneous studies in Jiangsu and Zhejiang Provinces, collectively indicating that non-occupational pesticide poisoning—particularly accidental and intentional exposures—represents a widespread and persistent burden across eastern China [13]. Substantial disparities in CFR were observed across etiologic subgroups: occupational poisoning exhibited the lowest CFR (5.1%), attributable to structured workplace surveillance systems enabling rapid identification and prompt referral to tertiary care facilities [14]. In contrast, accidental poisoning (CFR = 21.3%) and suicidal poisoning (CFR = 28.6%) demonstrated markedly elevated fatality risks—factors likely driven by treatment delays (median pre-hospital interval: 4.2 h), limited access to advanced life support in rural primary hospitals, and patient-specific comorbidities including cardiovascular disease and chronic respiratory conditions. The high incidence and disproportionate mortality of non-occupational poisoning not only strain regional healthcare resources—evidenced by prolonged ICU stays and increased demand for antidotes—but also generate profound socioeconomic consequences, including long-term disability, household financial catastrophe, and intergenerational impacts on child education and caregiver employment.

Age demonstrated a robust and non-linear association with both case distribution and mortality risk. The overall case count increased progressively with advancing age, peaking among individuals aged 65–74 years (28.3% of total cases), a pattern consistent with epidemiological trends reported in Quzhou City [10]. This age gradient reflects the demographic reality of rural population aging in Zibo, where middle-aged and elderly residents constitute the primary agricultural labor force—and thus the principal cohort exposed to pesticides—while concurrently experiencing diminished health literacy, lower adherence to protective practices, and reduced access to timely, high-quality emergency care [15, 16]. Notably, children under 15 years accounted for only 4.1% of all cases yet represented 12.1% of accidental poisoning incidents, underscoring the critical vulnerability of this group to unintentional household exposures and reinforcing the urgent need for evidence-based interventions targeting safe pesticide storage, child-resistant packaging, and caregiver education. With regard to suicidal poisoning, two distinct high-risk age strata emerged: adults aged 25–55 years (58.7% of suicidal cases) and older adults ≥ 65 years (29.4%). Among the former, socioeconomic stressors—including unemployment, debt burden, and marital discord—were predominant drivers; among the latter, risk was strongly associated with social isolation, inadequate community mental health support, and comorbid chronic conditions such as depression, hypertension, and dementia [17–25].

In the context of gender distribution, the number of male cases was slightly higher than that of female cases, which is associated with their greater participation in agricultural production and exposure to pesticides [1, 2, 10, 26]. Logistic regression analysis indicated that females faced a higher risk of death (OR = 1.69, 95%CI: 1.08–2.72). This gender disparity might be related to physical differences in pesticide metabolism, as well as different exposure scenarios and help - seeking behaviors between genders.

Pesticide poisoning exhibited a distinct seasonal distribution, with cases concentrated from March to October. This is in line with the busy agricultural production seasons, during which the increased use of pesticides and the high - temperature - induced pesticide volatilization collectively elevate the exposure risks [27, 28], offering a clear direction for seasonal prevention work.

Regional disparities were also noted. Linzi District and Zichuan District registered the highest number of cases, attributable to their agriculture - dominated industrial structure and the frequent exposure of residents to pesticides. The relatively high CFR in Huantai District and Yiyuan District might be related to the local economic conditions and the limited medical resources, which could result in delayed treatment and unfavorable prognosis. Consequently, it is necessary to develop differentiated prevention and treatment strategies based on regional industrial characteristics and medical circumstances.

Organophosphates were the primary causative agents of pesticide poisoning (Fig. 6). Their extensive application in agriculture, high acute toxicity, and easy accessibility result in a high risk of both accidental and intentional poisoning.

**Fig. 6:**
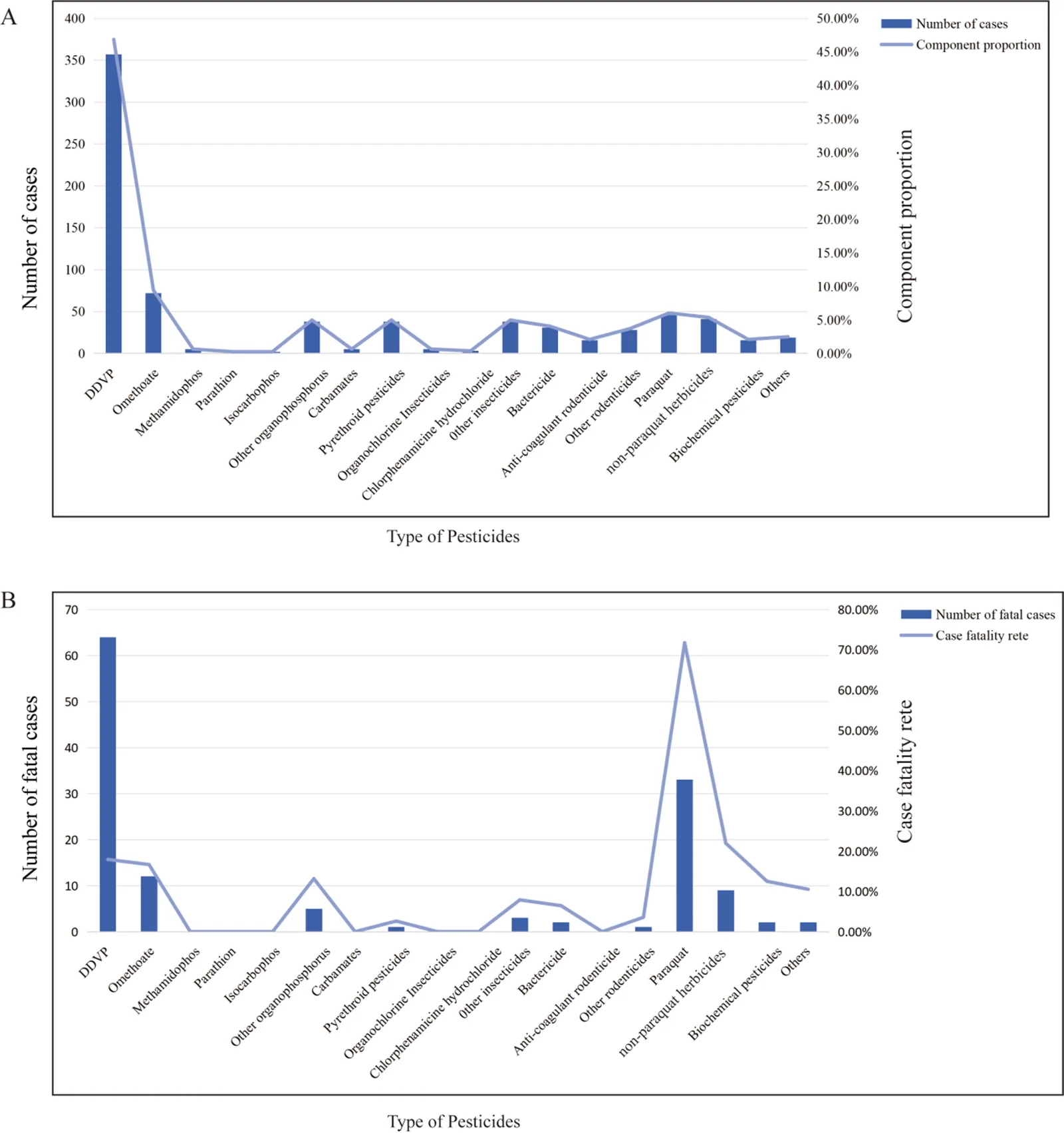
Types of Pesticides Implicated in Poisoning Cases in Zibo City, 2014–2023: (**A**) Case Count and Proportion (%); (**B**) Fatal Case Count and Case-Fatality Rate (%)

Multivariate regression identified age, poisoning type, and pesticide category as crucial predictors of death. Individuals aged 45 and older exhibited significantly increased mortality risks, potentially attributable to the decline in physical function and delayed seeking of medical assistance. Suicidal poisoning was associated with a substantially higher risk of death compared to occupational poisoning, highlighting the significance of interventions for non - occupational exposure and psychological counseling. Herbicides and other pesticide types were correlated with a higher fatality rate than insecticides, which can be related to their toxic mechanisms and treatment challenges, providing references for pesticide supervision.

Based on the aforementioned results, we propose targeted suggestions for the prevention and control of local pesticide poisoning. Firstly, conduct regular publicity and training during the farming seasons, promote the use of low - toxic pesticides, and reduce poisoning resulting from misuse. Secondly, enhance market supervision of highly toxic pesticides to control risks at the source. Thirdly, implement targeted education and psychological counseling for high - risk groups, including the elderly, children, and young adults. Fourthly, improve the emergency treatment capacity of primary medical institutions to shorten the treatment delay and reduce the mortality rate.

This study encompasses several limitations. Firstly, the data were extracted from a unified monitoring system, and cases treated in informal medical institutions were excluded, which might lead to an underestimation of the overall incidence. Secondly, the research was confined to Zibo City; hence, the results cannot be directly extrapolated to other regions. Thirdly, the small sample size of certain subgroups could impact the stability of relevant results. Fourthly, only municipal - level population data are accessible in the Zibo Statistical Yearbook, rendering it infeasible to calculate district - level incidence rates. Future studies should broaden the scope of data collection and research. Further follow - up investigations are also necessary to validate the effectiveness of the proposed intervention measures.

## Supplementary Information

Supplementary Material 1.

## Data Availability

The data that support the findings of this study are available from the National Occupational Disease and Occupational Health Information Monitoring System, and these data are not publicly available.
